# Dynamic Turning of a Soft Quadruped Robot by Changing Phase Difference

**DOI:** 10.3389/frobt.2021.629523

**Published:** 2021-04-22

**Authors:** Hiroaki Tanaka, Tsung-Yuan Chen, Koh Hosoda

**Affiliations:** Adaptive Robotics Laboratory, Graduate School of Engineering Science, Osaka University, Toyonaka, Japan

**Keywords:** quadruped robot, soft robotics, pneumatic artificial muscle, body-environment interaction, dynamic turn

## Abstract

Dynamic locomotion of a quadruped robot emerges from interaction between the robot body and the terrain. When the robot has a soft body, dynamic locomotion can be realized using a simple controller. This study investigates dynamic turning of a soft quadruped robot by changing the phase difference among the legs of the robot. We develop a soft quadruped robot driven by McKibben pneumatic artificial muscles. The phase difference between the hind and fore legs is fixed whereas that between the left and right legs is changed to enable the robot to turn dynamically. Since the robot legs are soft, the contact pattern between the legs and the terrain can be varied adaptively by simply changing the phase difference. Experimental results demonstrate that changes in the phase difference lead to changes in the contact time of the hind legs, and as a result, the soft robot can turn dynamically.

## 1. Introduction

Quadruped locomotion is more stable than bipedal locomotion and more adaptive than wheeled locomotion to the terrain (He and Gao, 2020). However, if each leg is controlled to follow a given trajectory based on force or touch information, the movement of the robot cannot be very fast and adaptive. Thus, Compliance of each leg should to be a key to resolve the problem. Spröwitz et al. (2013) developed a robot Cheetah-cub, a quadruped robot with compliant legs. Recently, to realize fast and adaptive locomotion, some studies (e.g, Hyun et al., 2014) have proposed using low-reduction gears and impedance control. Pneumatic artificial muscles are also good candidates for realizing fast and adaptive quadruped locomotion, because they have natural compliance and can react against the external forces without a time delay (Narioka et al., 2012). However, these muscles have a large control latency, and it is very difficult to be control them precisely. To change the robot behavior, for example, changing the direction of the movement, we have to develop a different method for controlling the actuators.

This study describes a quadruped robot driven by pneumatic artificial muscles, and investigates the turning behavior by changing the phase difference among the legs. Pneumatic muscles are light; however, they can generate relatively large power. The compliance of the actuator absorbs the impact that is associated with landing without feedback control. In addition, the artificial muscles can be arranged similar to the muscles of animals, which may provide bio-inspired design guidelines for the hardware and control. The tunable compliance of the actuator enables multi-modal locomotion (Hosoda et al., 2008). It is even possible for a musculoskeletal robot to generate a gait pattern, without needing any computational resources (Masuda et al., 2020). However, it is difficult to control them precisely to change the behavior of the robot, because they are quite nonlinear and have a large control latency.

The target behavior of this paper is turning, which has been a complex locomotion task. In static locomotion, the robot can turn by changing the trajectory of its legs (Chan and Liu, 2016). To realize dynamic turning, however, it is necessary to analyze the dynamic effects such as centripetal force (Tsujita et al., 2005). Some researchers have proposed the application of computational techniques, such as optimization or learning methods (Bledt et al., 2018; Fahmi et al., 2019; Hwangbo et al., 2019), without explicitly dealing with the dynamic effects. The RHex robot utilizes hardware compliance for dynamic turning. The robot turns dynamically by changing the phase difference between the left and right sets of legs (Haldane and Fearing, 2014). Haldane et al. analyzed the turning moment that generates roll oscillation, leading to the turning motion of the robot in the simulation. This paper also utilizes phase difference for a pneumatically driven quadruped robot. We propose a simple controller for a quadruped robot that changes the phase difference among the legs, and analyze the contact pattern of the soft legs for dynamic turning.

The remainder of this paper is organized as follows. First, we describe the design of a quadruped robot driven by McKibben pneumatic artificial muscles. Each leg has two degrees of freedom (DOF) and is driven by a fixed-valve pattern. The diagonal legs are in phase, and the phase difference between the left and right legs can be changed. Then, we experimentally investigate whether the turning can be controlled by changing the phase difference. We recorded the contact patterns of the leg during the experiments, and found a relation between the patterns and turning motion. The main contributions of this paper are (1) developing a pneumatically driven soft quadruped robot that can turn, (2) determining the relationship between the contact patterns of the legs and turning behavior, and (3) finally, experimentally realizing the turning of the robot experimentally.

## 2. Hardware Design

During locomotion without slipping, a robot is strongly restrained on the ground and receives a strong ground reaction force (GRF). A change in the contact timing of the leg can cause the robot to fall over, depending on the hardware design. For example, unlike a conventional motor-driven robot, a soft robot can contact the ground adaptively without falling over, even if the contact timing is changed (Rosendo et al., 2014). Therefore, we focus on softness and develop a soft quadruped robot named “PneuHound.” In this section, we introduce the hardware design of the developed robot.

### 2.1. Mechanical Design of Quadruped Robot

**Figure 1:**
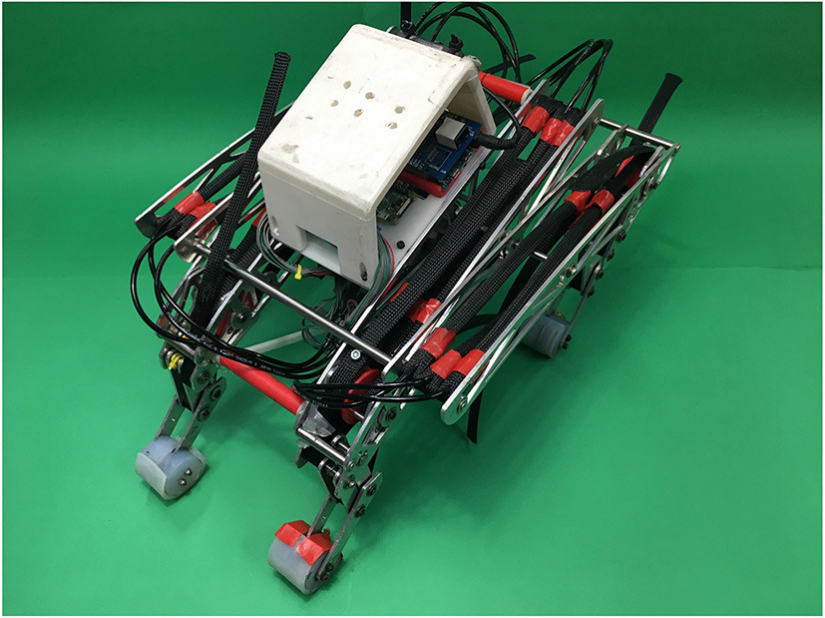
Pneumatic quadruped robot “PneuHound.” The main objective of this robot is to perform dynamic turning by changing the phase difference among its legs.

Figure 1 provides an overview of the design of “PneuHound.” The length, width, height, and weight of the whole body are 500, 300, 300 mm, and 3.5 kg, respectively. The main structure is composed of aluminum components. Air and electrical power are supplied externally. More detailed information about the design is provided in Table 1.

**Table 1:** Key properties of PneuHound.

**Property**	**Value**
Length × width × height	500 × 300 × 300 mm
Total weight	3.5 kg
Fore leg weight	0.24 kg
Hind leg weight	0.26 kg
Number of valves	8
Number of muscles	12
*L*1 segment length in fore legs	55 mm
*L*2 segment length in fore legs	52 mm
*L*2 segment length in fore legs	70 mm
*L*1 segment length in hind legs	65 mm
*L*2 segment length in hind legs	64 mm
*L*3 segment length in hind legs	70 mm

**Figure 2:**
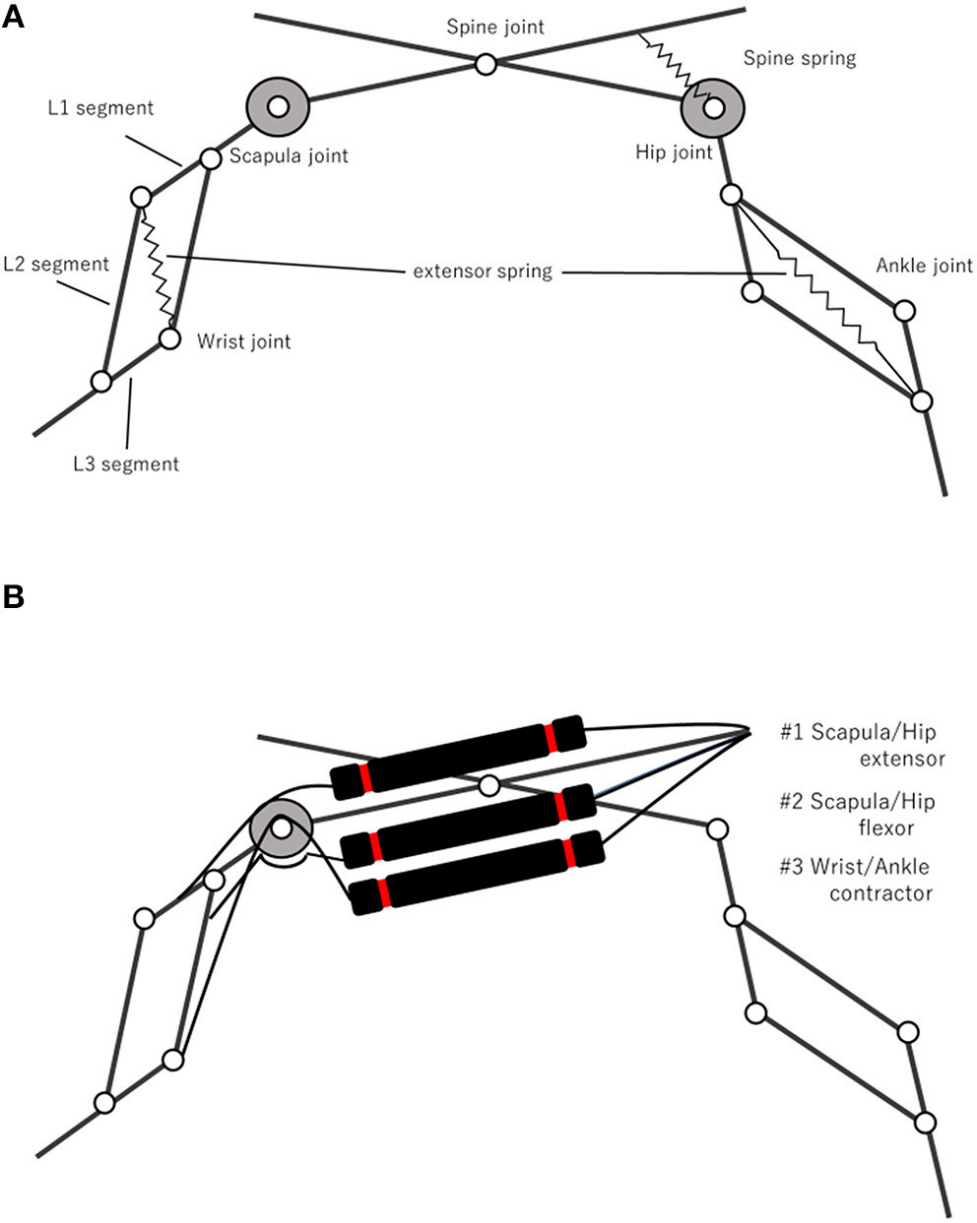
Musculoskeletal structure of “PneuHound.” Each leg, consisting of four links and four joints, one of which is constrained by a pantograph mechanism, has two DOFs. Passive springs are provided on the spine joint and pantograph mechanisms **(A)**. There are three muscles for each limb; #1 Scapula/Hip extensor, #2 Scapula/Hip flexor, and #3 Wrist/Ankle contractor, and each pantogragh leg is composed of an *L*1segment, *L*2segment, and *L*3segment **(B)**.

The musculoskeletal structure of “PneuHound” is shown in Figures 2A,B. The robot has nine DOFs in total: each leg has two DOFs, and the spine has one DOF. The leg design is based on those reported in previous studies (Spröewitz et al., 2011; Narioka et al., 2012). Each leg has a pantogragh structure with four links and four joints, and a tension spring is provided on the diagonal of the pantograph structure. The fore and hind legs can be extended with springs to a maximum of 65 and 70 mm, respectively. Due to the difference between the lengths of the fore and hind legs, the spring constants are different: 2.4 and 2.9 N/mm for the fore and hind legs, respectively.

To manipulate the leg, the robot drives nine joints: scapula and wrist joints in the fore legs, hip and ankle joints in the hind legs, and the spine joint (Figure 2A). The scapula and hip joints are antagonistically driven by two muscles, the wrist and ankle joints are retracted by a muscle and extended by a spring, and the spine joint is driven only by a spring (Figures 2A,B). The moment arm of the muscles driving the scapula and hip joints can be adjusted through the diameter of the pulley, which is set as 40 mm. In contrast, the moment arm of the wrist and ankle flexors is very small (5 mm); this ensures that the flexors can shorten the leg while minimizing the influence of the muscle driving the scapula and hip joints.

For running dynamically without slippage, the robot requires high ground fiction. Hence, we install rubber pillars on the toes. The radius and height of the rubber pillars are 17.5 and 37 mm, respectively.

### 2.2. Pneumatic Actuator and Control Architecture

Animals possess biological muscles, which provide a high output force with compliance to enable locomotion. To impart softness to the robot, we use McKibben pneumatic artificial muscles, which are similar to biological muscles, as the actuators. For such artificial-muscle-type actuators, the principle of actuation is that supplying air to the muscle generates a contraction in the muscle, while exhausting the same air relaxes the muscle. The compliance offered by the actuator is proportional to the contraction, and the force provided by the actuator depends on the internal pressure and the muscle deformation, as shown in the following equation (Klute et al., 2002):

(1) $$\begin{array}{r} F\propto \frac{P_{air}}{\Delta /L_{0}}, \end{array}$$

where *F* is the force, *P*_*air*_ is the internal pressure, *L*_0_ is the relaxed length, and Δ is the deformation of the muscle. The actuators are made from a rubber tube with an 8-mm diameter, 1-mm thickness, and 200-mm length, covered with a polyester exterior braid having an 11-mm diameter. The actuators are connected to a valve through a tube with a 4-mm diameter.

**Figure 3:**
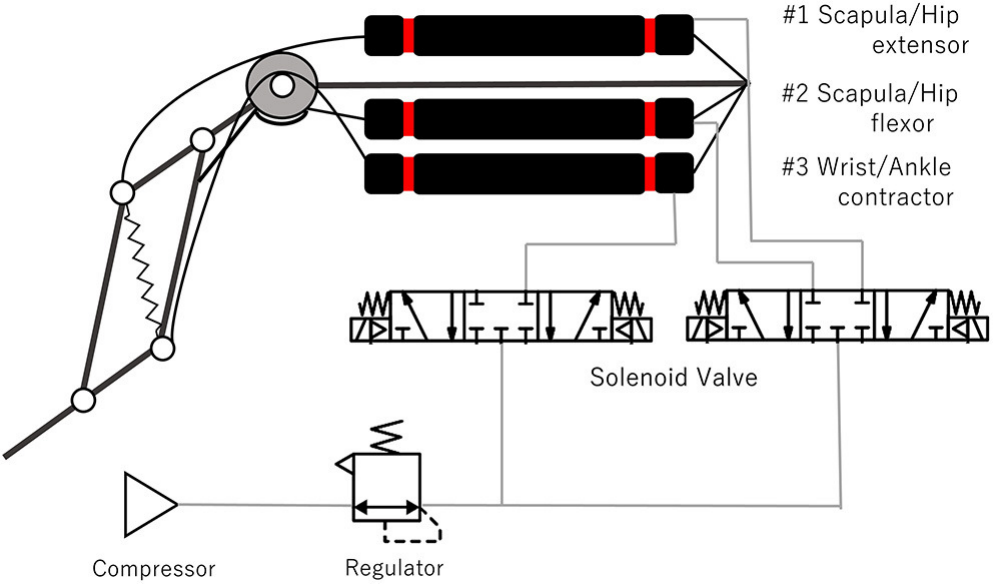
Pneumatic actuators and pneumatic system for driving one leg.

Figure 3 shows the pneumatic system for driving one leg with three actuators. Two solenoid valves (5 ports and 3 position types, SYJ3340-6L, SMC Co.) are used for each limb. One of the valves supplies compressed air to both the extensor and the flexor driving the scapula and hip joints, while the other valve supplies compressed air to the contractor driving the wrist and ankle joints. The two actuators driving the scapula and hip joints constitute the antagonistic muscles. However, only one valve is connected to these muscles, and hence, the joint compliance can't be modulated by varying the pressure of the antagonistic muscles as achieved in previous studies (Hosoda et al., 2008; Takuma et al., 2008). The supply air pressure was 0.54 MPa when the turning experiment was conducted. A micro–controller (Arduino–Due) with the custom amplifier board is used for valve control.

## 3. Controllers for Dynamic Locomotion

To achieve dynamic turning for a rigid quadruped robot, previous studies focused on controlling the phase difference among the legs to adjust the imbalance between left and right movements (Tsujita et al., 2005). This was only for compensating the imbalance, not for generating turning motion. In contrast, we control the phase difference among the legs to generate turning motion, based on a previous study (Haldane and Fearing, 2014). To design the locomotion controller, we divide it into one-leg and inter-limb controllers. In this section, we explain the design of each controller.

**Figure 4:**
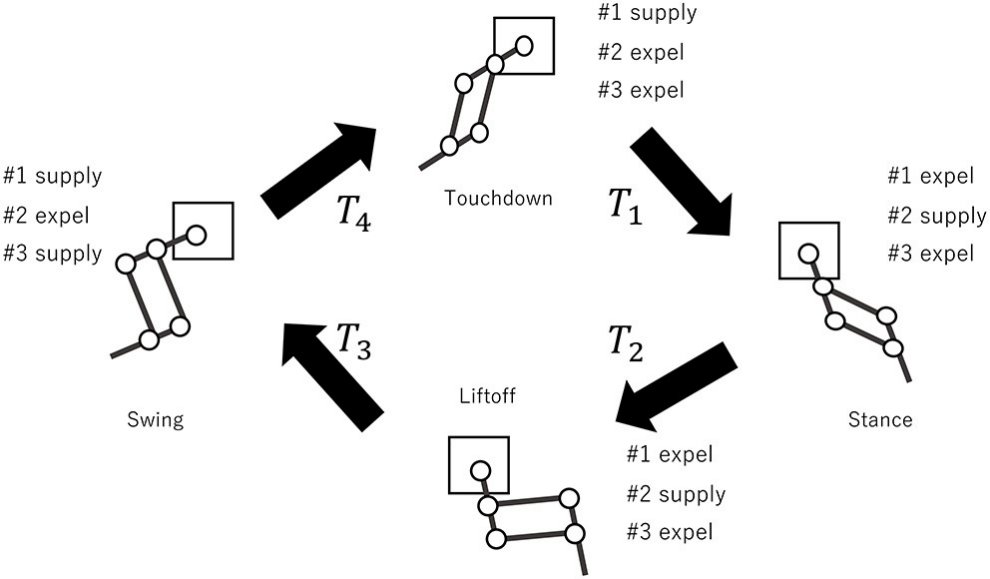
Proposed one-leg controller. The cyclic motion of the leg is divided into four phases: touchdown phase for *T*_1_ ms, stance phase for *T*_2_ ms, lift-off phase for *T*_3_ ms, and swing phase for *T*_4_ ms.

To generate the motions of the right hind leg (RH), right fore leg (RF), left hind leg (LH), and left fore leg (LF), we design the one-leg controller dividing the cyclic motion of a leg into four phases (Figure 4). The four phases are touchdown, stance, lift-off, and swing. To execute these phases, the valve–opening and -closing times, which determine the actuation pattern of muscles #1, #2, #3 (Figure 2B), are determined. For the touchdown phase, muscle #1 is supplied with air, while muscles #2 and #3 are expelled for *T*_1_ ms. For the stance phase, muscle #2 is supplied, while muscles #1 and #3 are expelled for *T*_2_ ms. For the liftoff phase, muscles #2 and #3 are supplied, while muscle #1 is expelled for *T*_3_ ms. For the swing phase, muscles #1 and #3 are supplied, while muscle #2 is expelled for *T*_3_ ms. Consequently, the period of the leg motion is calculated as *T* = *T*_1_ + *T*_2_ + *T*_3_ + *T*_4_ ms. We heuristically determined the parameters *T*_1_ ~ *T*_4_, such that the robot could run fast in the forward direction (i.e., *T*_1_ = 0.1*T, T*_2_ = 0.4*T, T*_3_ = 0.1*T, T*_4_ = 0.4*T*). Herein, the one-leg controller of each leg is denoted as the leg name followed by “controller” (for example, the one-leg controller of LF is denoted as “LF controller”).

**Figure 5:**
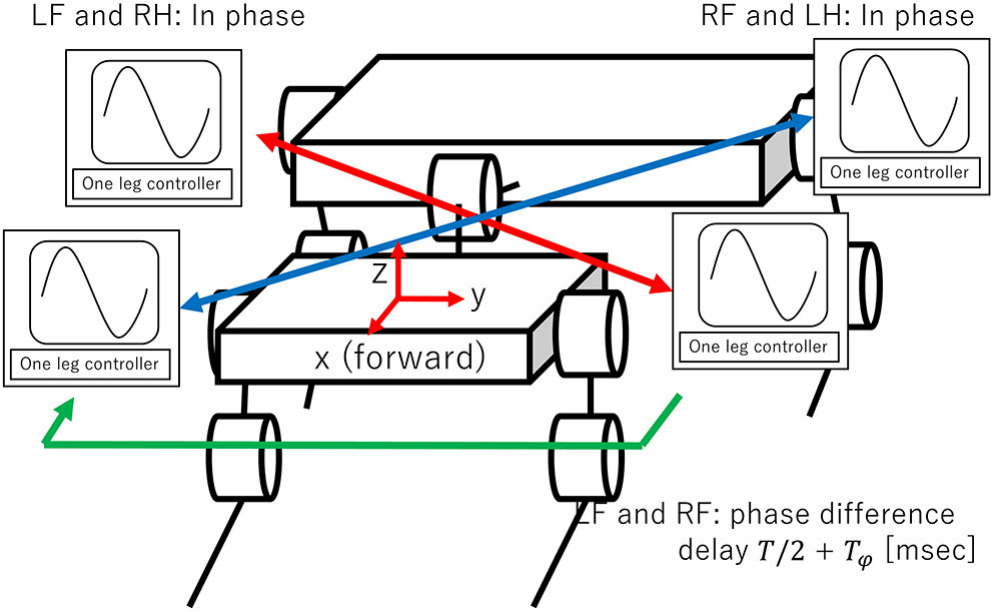
Proposed interlimb controller. The phase difference is set as follows: LF and RH controllers and RF and LH controllers are in phase. Phase difference between two diagonal pairs (LF and RH controllers and RF and LH controllers) can be changed by varying *T*_*ϕ*_.

Next, we design the inter-limb controller (Figure 5). The LF and RH controllers and the RF and LH controllers start at the same time. This means that the movements of the two diagonal pairs are in phase. To set the phase difference between these two diagonal pairs, the RF controller is started *T*/2 + *T*_*ϕ*_ ms after the LF controller. The phase difference can be changed from the trot gait (*T*_*ϕ*_ = 0) by varying the parameter *T*_*ϕ*_.

## 4. Experimental Setup

**Figure 6:**
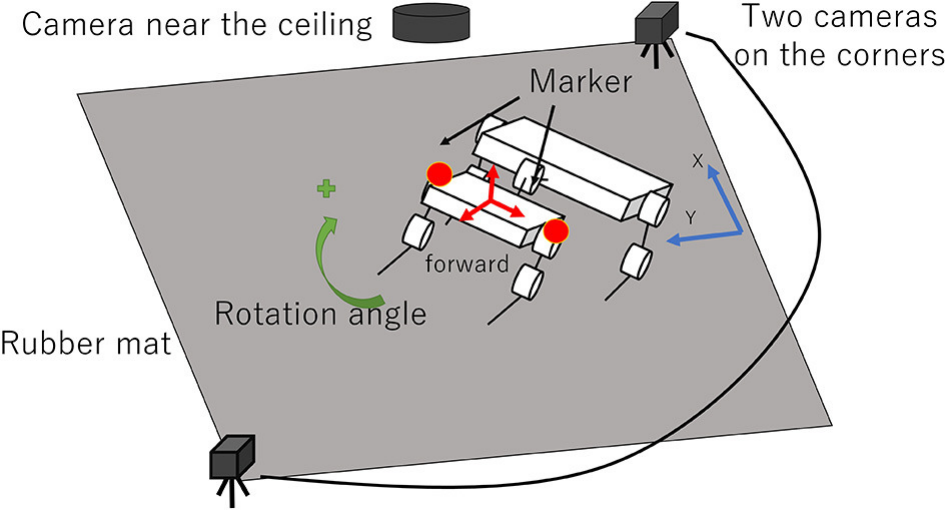
Experimental setup.

We used the experimental setup shown in Figure 6, which consists of the robot, a rubber mat, two cameras on diagonally opposite corners of the rubber mat, and a camera near the ceiling. The size of the rubber mat is 310 cm (L) × 180 cm (W). The two cameras on the corners of the rubber mat are synchronized, and their frame rate is 60 fps. The camera near the ceiling is 250 cm from the floor and captures the entire rubber mat; its frame rate is 30 fps. For a behavior analysis of the robot, we used image–processing software (Dipp-MotionV, DITECT, Inc.), and two makers are attached to the left and right sides at the front of the robot.

In this research, we define dynamic turning as follows: First, we define “dynamic locomotion” as locomotion with a period in which two or fewer legs contact with the ground. Next, “dynamic turning” is defined as the dynamic locomotion with a turning motion.

In the following experiments, we observe four quantities: velocity, turning rate, duty ratio (the ratio of stance period divided by cycle period), and the ratio of the period that two or fewer legs contact with the ground divided by cycle time. The velocity is calculated with reference to the midpoint of the two markers on the robot. The turning rate is calculated using the two markers; the angle when turning right from the initial posture is considered as positive. The impulse received from the ground can be estimated from the ground contact time and gives the robot propulsion. Therefore, we use duty ratio as a simplified indicator of propulsion and investigate the changes in this parameter according to the phase difference among the legs. Contact between the legs and the ground is identified from the videos recorded by the two synchronized cameras, and the duty ratio is calculated accordingly. To verify whether the locomotion of the robot is dynamic turning, the ratio of the period when two or fewer legs contact with the ground divided by cycle time is calculated. We conducted experiments by varying *T*_*ϕ*_ from 0 ms in increments of ±15 ms with *T* = 150, 300, 450.

## 5. Results

### 5.1. Velocity and Turning Rate in Dynamic Turning

**Figure 7:**
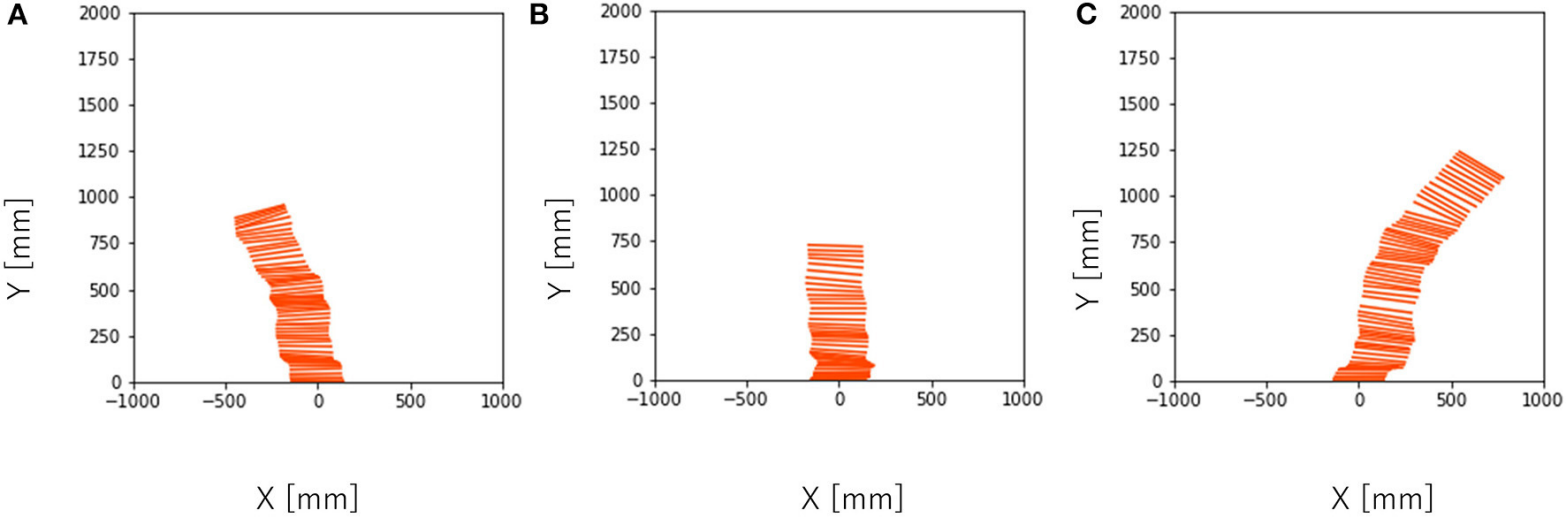
Turning behavior captured by the camera near the ceiling every 1/30 s with T = 300. **(A)** *T*_*ϕ*_ = −15, **(B)** *T*_*ϕ*_ = 0, **(C)** *T*_*ϕ*_ = 15.

**Figure 8:**
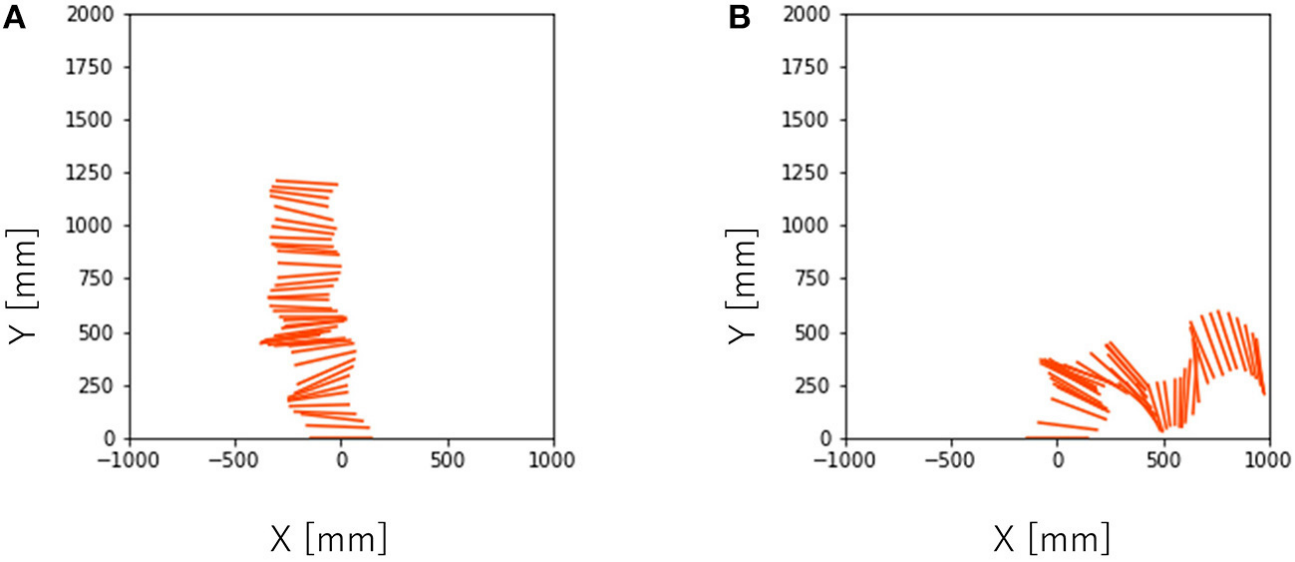
Turning behavior captured by the camera near the ceiling every 1/30 s with T = 450. **(A)** *T*_*ϕ*_ = 0, **(B)** *T*_*ϕ*_ = 15.

To investigate the effect of the phase difference among the legs on the dynamic turning, we conducted an experiment by varying *T*_*ϕ*_ where *T* = 150, 300, 450. With *T* = 150, the running was not successful because the supply time of the air was too short for the system to provide sufficient actuation force for running. With *T* = 300, the robot ran for *T*_*ϕ*_ = −30, −15, 0, 15, 30. Figure 7 shows the turning behavior of the robot with different parameters. With *T* = 450, the robot ran for *T*_*ϕ*_ = 0, 15. Figure 8 shows the turning behavior of the robot with different parameters.

**Figure 9:**
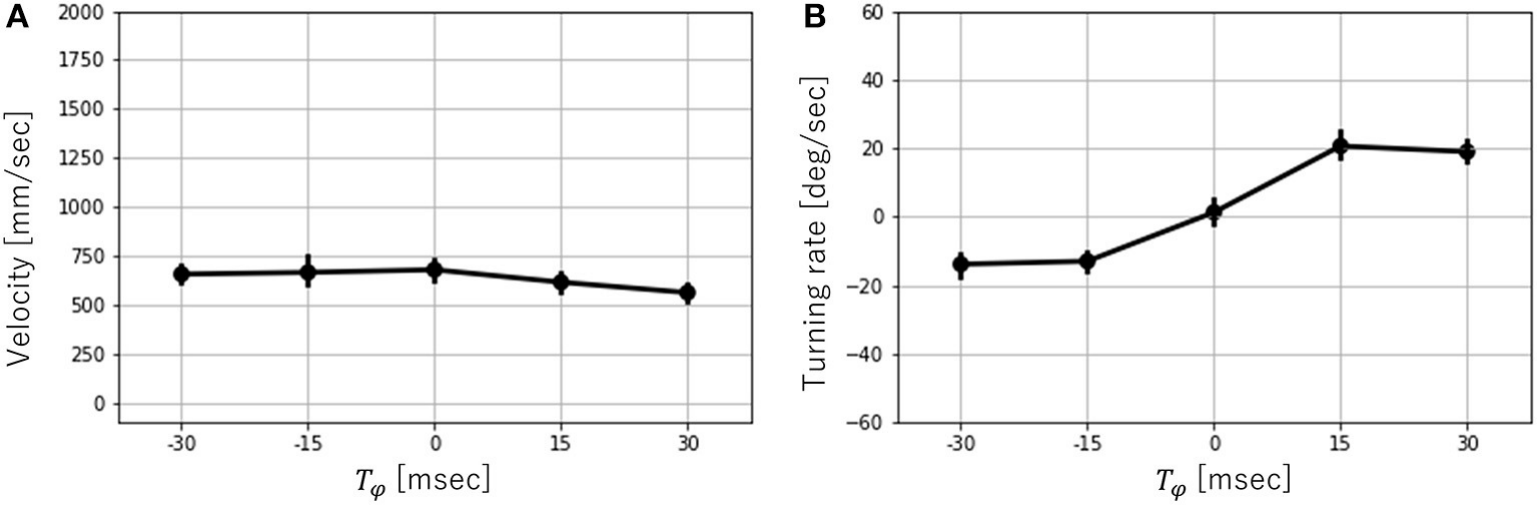
**(A)** Velocity vs. *T*_*ϕ*_ and **(B)** Turning rate vs. *T*_*ϕ*_ with *T* = 300. Error bars show one standard deviation.

**Figure 10:**
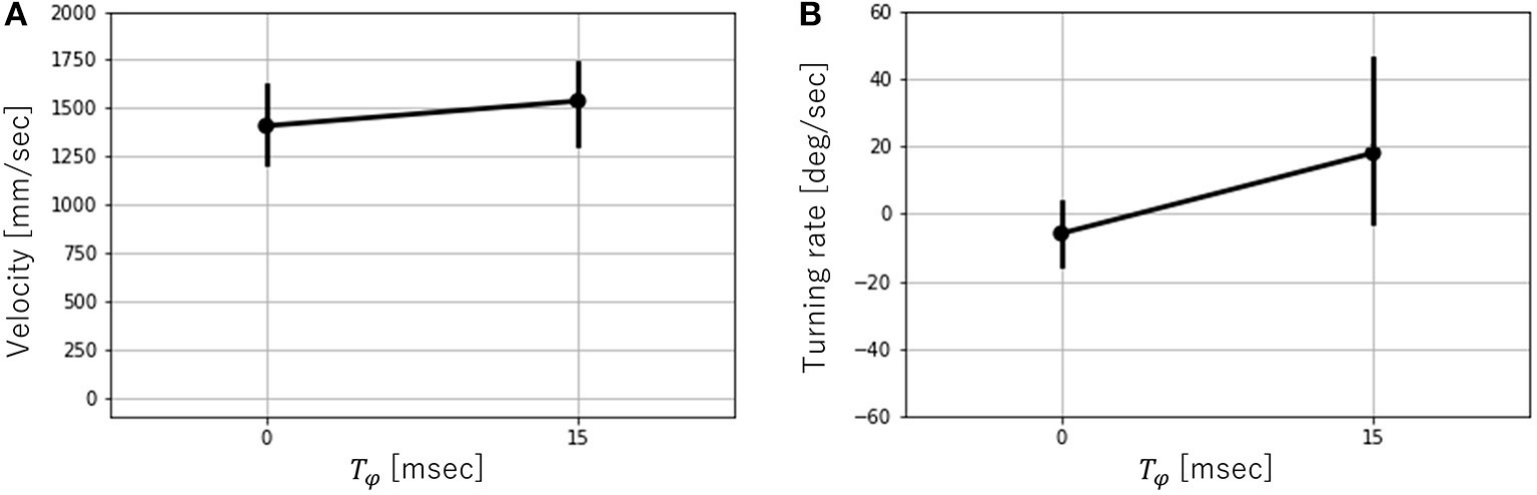
**(A)** Velocity vs. *T*_*ϕ*_ and **(B)** Turning rate vs. *T*_*ϕ*_ with *T* = 450. Error bars show one standard deviation. It is noted that the mean and the standard deviation are calculated using the data the robot successfully ran.

Next, the effects of *T*_*ϕ*_ on the velocity and turning rate were examined when the robot ran successfully (that is, *T* = 300, 450). Figure 9 shows the mean values and error bar of the velocity and turning rate with *T* = 300. A total of 10 trials were conducted for each parameter, and the average velocity and turning rate were obtained through the 2-s locomotion experiments. While the robot moved straight ahead with *T*_*ϕ*_ = 0, it turned right for *T*_*ϕ*_ > 0 and left for *T*_*ϕ*_ < 0. The velocity varied slightly depending on the value of *T*_*ϕ*_. In contrast, the turning rate varied significantly over the range of −15 ≤ *T*_*ϕ*_ ≤ 15. However, it did not change considerably over the ranges of −30 ≤ *T*_*ϕ*_ ≤ −15 and 15 ≤ *T*_*ϕ*_ ≤ 30. Next, the velocity and turning rate with *T* = 450 were investigated. Ten trials were conducted for each parameter, and the average velocity and turning rate were obtained through the 2-s locomotion experiments. However, only six trials were successful with *T*_*ϕ*_ = 0, and only seven trials were successful with *T*_*ϕ*_ = 15. Figure 10 shows the mean values and error bar of the velocity and turning rate with *T* = 450. It is noted that the mean and the standard deviation are calculated using the data in which the robot ran successfully. The velocity with *T* = 450 was higher, and had a larger variation than that with *T* = 300. The turning rate with *T* = 450 had a larger variation than that with *T* = 300, and showed a similar change with *T* = 300 between *T*_*ϕ*_ = 0 and 15.

### 5.2. Contact Pattern of Leg

**Figure 11:**
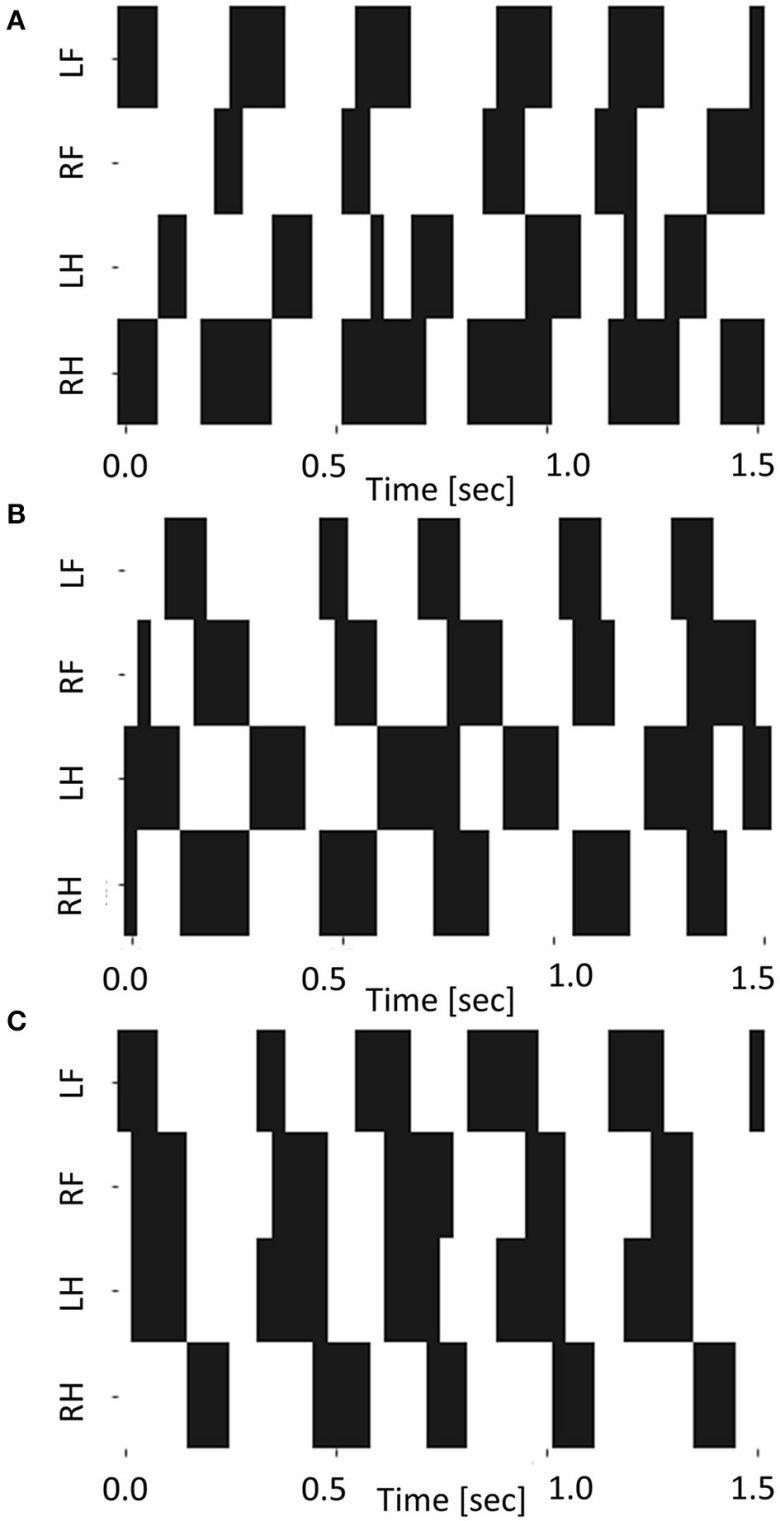
The gait diagram during 0.0–1.5 s for **(A)** *T*_*ϕ*_ = −15, **(B)** *T*_*ϕ*_ = 0, and **(C)** *T*_*ϕ*_ = 15.

**Figure 12:**
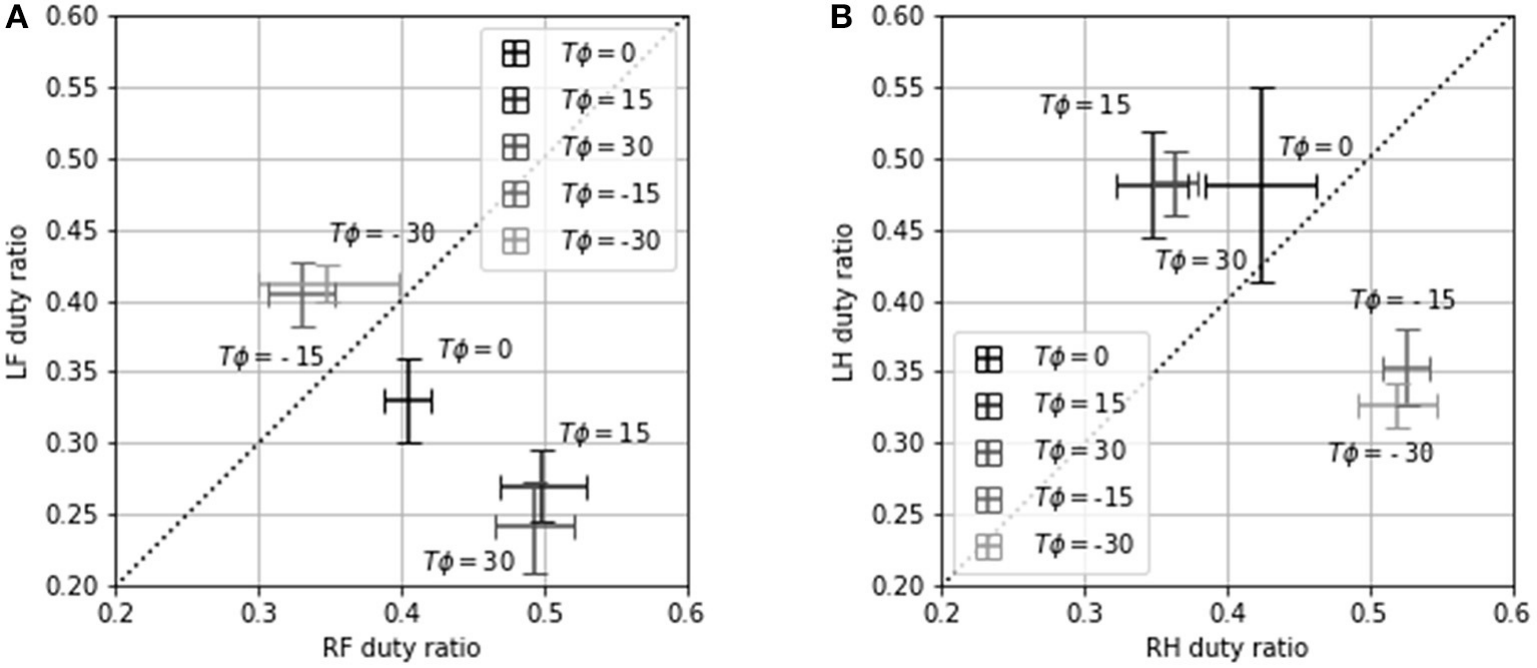
**(A)** LF duty ratio vs. RF duty ratio and **(B)** LH duty ratio vs. RH duty ratio for *T*_*ϕ*_ = −30, −15, 0, 15, 30. Error bars show one standard deviation.

In this experimental setup, we could capture the contact of the leg with *T* = 300. Hence, we conducted an experiment to investigate the dependence of the contact pattern of the leg on *T*_*ϕ*_ with *T* = 300. Table 2 shows the ratio of the period that two or fewer legs contact with the ground divided by cycle time with *T* = 300. This table shows that the robot locomotion is dynamic turning considering Figure 9 shows that the robot turned with *T* = 300. Figure 11 shows the gait diagram during 0.0–1.5 s with different values of *T*_*ϕ*_. In the gait diagram, the black regions represent the stance phase. The robot achieved different contact patterns by changing the value of *T*_*ϕ*_. The results showed that the robot turned dynamically because there was a phase in which two or fewer legs were in contact with the ground. Figures 12A,B shows the mean value and error bar of the duty ratio of the fore and hind legs. The line along which the duty ratios of the “left and right legs” are equal is also shown in the figure. Five trails were conducted for each parameter, and the average duty ratio was obtained through experiments involving more than 10 steps of locomotion.

**Table 2:** Ratio of the period that two or fewer legs contact with the ground divided by cycle time.

**Phase difference**	**Ratio**
*T*_*ϕ*_ = −30	0.80
*T*_*ϕ*_ = −15	0.86
*T*_*ϕ*_ = 0	0.74
*T*_*ϕ*_ = 15	0.86
*T*_*ϕ*_ = 30	0.89

From the experimental results, we observed a strong correlation between the duty ratio of the hind legs and the turning rate. For *T*_*ϕ*_ ≥ 15, the duty ratio of LH was higher than that of RH (Figure 12B). Consequently, the left propulsion was stronger than the right propulsion, causing the robot to turn right (Figure 9B). For *T*_*ϕ*_ ≤ −15, the duty ratio of RH was higher than that of LH, causing the robot to turn left. However, the relationship between the duty ratios of LF and RF was the opposite of the relationship between the duty ratios of LH and RH. The propulsion of the hind legs was observed to have a stronger influence on the turning of the robot than the propulsion of the fore legs. Additionally, the variation in the duty ratio was a similar to that in the turning rate: The duty ratio and the turning rate varied significantly over the range of −15 ≤ *T*_*ϕ*_ ≤ 15, but they did not vary considerably over the range of −30 ≤ *T*_*ϕ*_ ≤ −15 and 15 ≤ *T*_*ϕ*_ ≤ 30. This indicates that the contact pattern was not changed significantly upon changing the phase difference. As a result, the turning rate was not changed significantly either.

In addition, the experimental results show that the frequency of the periodic leg movement (that is, 1/T) changed the turning motion. Stable turning motion was generated with *T* = 300. When the frequency of the leg movements increased, the running was not successful. On the contrary, when the frequency of the leg movement decreased, the turning became unstable.

## 6. Discussion

We proposed a simple control method for dynamic turning of a quadruped soft robot driven by pneumatic artificial muscles as actuators. The experimental results show that the control parameter *T*_*ϕ*_ controls the direction of dynamic turning. Our results also indicate that when the gait deviates from trotting (*T*_*ϕ*_ = 0) due to changes in *T*_*ϕ*_, the contact pattern of the legs is changed, causing the robot to turn. However, the contact pattern does not change significantly depending on the size of the phase difference. Consequently, the turning rate does not change significantly either. In addition, the experimental results show that the frequency of the periodic leg movement changes the turning motion.

Haldane et al. reported that the phase difference between the left and right sets of compliant legs of a hexapod robot modulated the oscillations in height and roll angle. Additionally, they demonstrated that the roll oscillations enabled the robot to turn at high speeds through changes in the phase difference (Haldane and Fearing, 2014). In their study, to calculate the turning moment, the simulation setup parameters were simplified: the contact of the leg was defined only by the rotation angle of the motor. However, the interaction between the legs and the ground has to be carefully considered because various speeds and orientations are involved in the dynamic locomotion. Our study presents a significant finding: the contact pattern of a soft leg plays an important role in dynamic turning. In contrast, it seems that the roll oscillations confirmed in their study did not exist in our system because the roll posture data of our robot were not steady. The turning controllers of our study and their study were similar. However, because the main structure of our robot's body is different from that of their robot, the turning mechanism may be different.

In previous studies, the turning direction of motor-driven robots have been precisely controlled using kinematic approaches. For example, generating a three-dimensional motion by increasing the DOF of the legs improves turning maneuverability (Estremera and de Santos, 2005; Kimura et al., 2007; Raibert et al., 2008). In this study, the trajectory of each leg in the air was kept unchanged since we aimed to investigate the effect of the phase difference among the legs. We believe that integration of the kinematic method with our control method for soft robot legs presents an important topic for further research on precise control of the turning direction with a simple controller.

Our research has three limitations. First, is that in this experimental setup, we could not capture the contact pattern of the leg with *T* = 450 because the robot turned in various directions. Therefore, we cannot confirm the effect of unstable turning on the contact of the legs. Second, we did not verify that the same simple controller would not produce turning in a non-soft robot. In this experimental setup, it was extremely difficult to control the stiffness of the leg for a quadruped robot. Third, the structure makes a different turning mechanism from the relevant study (Haldane and Fearing, 2014). We intend to investigate more about these three issues in the near future.

## 7. Conclusion

In this study, we demonstrated dynamic turning of a soft quadruped robot with a simple controller. Our findings show that a soft body simplifies a controller design even for complex tasks. We showed that a change in phase difference leads to a change in the duty ratio of the hind legs, enabling the robot to turn dynamically. Further investigation of the softness of quadruped robots can elucidate the complex interaction between the robot and the terrain, enabling the development of a specialized control method for such robots.

## Data Availability Statement

The original contributions presented in the study are included in the article/Supplementary Material, further inquiries can be directed to the corresponding author/s.

## Author Contributions

HT: conception and design, development, analysis and interpretation, manuscript writing, and final approval of the manuscript. T-YC: construction of the robot and final approval of the manuscript. KH: development of fundamental concepts, project guidance, and final approval of the manuscript. All authors contributed to the article and approved the submitted version.

## Conflict of Interest

The authors declare that the research was conducted in the absence of any commercial or financial relationships that could be construed as a potential conflict of interest.
